# The Primary Care-Video Intervention Therapy for Growth-Vulnerable Infants. A Case Study

**DOI:** 10.3390/ijerph17051796

**Published:** 2020-03-10

**Authors:** Chiara Sacchi, Sergio Facchini, George Downing, Alessandra Simonelli

**Affiliations:** 1 Department of Developmental and Social Psychology, University of Padova (Italy), 35131 Padova, Italy; alessandra.simonelli@unipd.it; 2 Pediatric Primary Care Unit, National health Service (Italy), 33080 Pordenone, Italy; serfac.pn@gmail.com; 3 Clinical Faculty, Salpetriere Hospital and Paris University VIII (France), 75000 Paris, France; george.downing2@gmail.com

**Keywords:** parenting, infant mental health, primary care, video-feedback, Small for Gestational Age

## Abstract

Perinatal growth vulnerability (e.g., Small for Gestational Age, SGA) poses the goal to not overlook subtle developmental susceptibilities and their impact on the parent–infant relationship. In this case study, we examined the application of a video-feedback intervention program to support parenting, the Primary Care-Video Intervention Therapy (PC-VIT), specifically developed to fit pediatric care characteristics. The case presentation details the principal steps of the intervention with the family of an SGA infant from birth up to toddlerhood. Findings for this family highlighted initial worries about the infant’s growth. Along sessions, PC-VIT held maternal anxiety and sustained parents’ abilities to recognize and talk about the infant’s developmental skills and regulatory abilities. The PC-VIT shows the powerful opportunity to limit the impact of infant growth vulnerability on the parent–child relationship and socio-emotional development. Pediatricians can prevent vulnerable developmental milestones from clinical outcomes by implementing timely and effective strategies embracing mental health and parenting-related issues.

## 1. Introduction

Perinatal growth vulnerabilities (i.e., prematurity, low birth weight, low-to-moderate perinatal risk) expose child neurodevelopment and behavioral growth with potential consequences for parental attitudes and parenting behaviors [1]. Families with growth-vulnerable infants experience high levels of irritability and parenting difficulties during the first years of the child’s life that might disrupt the establishment of a healthy parent–child relationship [2]. Studies on preterm samples observe that a scarcity of communicative signals on the infant side challenges parents’ understanding of infant behaviors and make them activate compensatory responses [3,4]. Such compensatory behaviors, like excessive scaffolding, can be highly adaptive supporting infant initiatives, but can eventually result into intrusive and non-attuned parenting behaviors [5].

Among growth vulnerabilities, Small for Gestational Age (SGA) is a birth outcome classification describing newborns delivered at a birth weight below the tenth percentile for gestational age in their normal distribution reference curve. Being born SGA constitutes a risk factor for developmental impairments (i.e., cognitive and motor), emotional behavioral problems, and later health diseases [6]. Despite a lack of studies specifically focused on SGA socio-emotional development, a few data have pointed out early signs of atypical socio-emotional competences. Compared to normal birth weight peers, SGA newborns show poor use of environmental stimuli [7] and higher negative affects, along with difficulties in orientating to social and non-social environments [8]. In addition, significant delays have been observed in adaptive behaviors and social interactions domains in toddlerhood. In terms of parenting experiences, greater maternal intrusiveness along with infant passivity during interactive exchanges have been reported in SGA mother–infant dyads [9]. Besides, such parents are often confronted with distressing information about fetal and offspring size [10], so that maternal representations might be affected by a potential sense of inadequacy to provide the fetus with a growth-promoting inner antenatal environment resulting into offspring low birth weight. Such representations likely affect the mother’s emotional state, the engagement with the baby and her attitudes and behaviors toward parental care. Overall, in SGA parent–infant dyads, both parental difficulties and infant social vulnerability appear to potentially impact the establishment of a healthy, safe, and nurturing parent–child relationship, and thus infant socio-emotional growth. Several approaches have proved the efficacy of sustaining early parent–child interactions as a means to protect and boost socio-emotional growth [11,12]. Indeed, parenting is an excellent port-of-entry for early interventions directed to foster child development, and birth is the unique window for supporting parenting, since newborn survival emerges as the first parental concern, making the family system plastic and open to molding forces [13]. Unfortunately, in the absence of preterm delivery and/or high-perinatal risk, SGA infants are very unlikely to receive tailored follow-up care, so that their socio-emotional vulnerability and its effect on parenting might remain overlooked up to the onset of later emotional and behavioral problems and mental-health outcomes [14]. Consequently, growth vulnerabilities like SGA birth pose the clinical challenge of properly monitoring infant psychical development, without overlooking the subtle socio-emotional susceptibility and its potential impact on the parenting.

During the first year of a child’s life, pediatricians visit infants with their families earlier and more often than any other health professionals, with the great advantage that parents value that relationship and feel comfortable in openly discussing their concerns. Besides, infant’s physical growth represents a primary index of health and its assessment constitutes an essential part of pediatric care [15], giving the chance to explore parental mental representations surrounding a child’s growth. Pediatric consultations represent a relational context for parents to share a focus on infant healthy development and discuss their difficulties as parents. Therefore, primary care might constitute a timely setting to provide and promote an integrated view of infant mental and physical health, as well as to implement effective strategies nurturing a healthy and secure parent–child relationship.

### 1.1. Video-Feedback Interventions

Among early strategies to supporting parenting, Video Feedback (VF) is a powerful therapeutic tool, guiding the parents to analyze and reflect on video clips of their own interaction with the child. Evidence of VF effectiveness in early mother–child intervention is largely documented [12,16,17] and several applications across therapeutic modalities are known [18,19,20]. In its application to parenting, usually video-feedback is part of a multimodal approach also including instruction, therapeutic counseling, and other forms of support. Some approaches use a short series of sessions with specific themes designated to each session [21,22]. Other approaches base the choice of each session theme upon the particular case [23,24,25,26,27,28].Video-feedback for supporting parenting can be used for simultaneous purposes: (i) To aid parents to better notice and identify children’s cues; (ii) to recognize and perhaps change parental behaviors; (iii) to better hypothesize the motivational roots of the child’s behaviors. Overall, parents’ experience of observing themselves in the video aids achieving a more realistic perspective on their relationship with their child [29]. In particular, they become more aware of their own reactions and are supported in better hypothesizing the motivational roots behind the child’s behaviors.

### 1.2. The Video Intervention Therapy and Primary-Care Video Intervention Therapy

Among video feedback interventions, Video Intervention Therapy [30] is a mentalization-based cognitive-behavioral methodology. Beyond classical behavior-oriented techniques, it draws on mentalizationeliciting, and other techniques developed within VIT itself [30,31]. Mentalization refers to the capacity to understand oneself and others in light of mental states [32]. In the specific context of parenting, it represents the parental attitude of making sense of the child’s behaviors as an expression of internal emotional and mental states. It is a powerful predictor of parent–child attachment security, since parents are more likely to respond sensitively to a child’s signals when they can understand the meaning and intentions of the child’s behaviors [33]. Besides, the parental capacity to treat the child as a psychological agent positively impacts the child’s socio-cognitive development, stimulating his or her own mentalizing capacity, autonomy, and self-regulation [34]. In VIT sessions, mentalizing techniques are used to identify difficulties in the parent–child relationship and intervene to improve the connection between the parent and child, teaching parents to recognize and understand their child’s mental states through the support of the video.

The Primary Care-Video Intervention Therapy (PC-VIT [35,36]) program is an adaptation of the original VIT [30], specifically developed to suit characteristics of a pediatric setting. Indeed, during the pediatric consultations, pediatricians observe a widespread range of early parent–infant interactive exchanges that provide a wealth of relevant cues about socio-emotional development and the parenting. Besides, the pediatric context excels in being a suitable setting to address parenting and mental-health issues surrounding atypical and/or vulnerable infant growth. The specificity of PC-VIT is that each session focuses on the characteristic developmental challenge faced by the family during the first year of a child’s life at different time points corresponding to the well-baby visit. Through the VF, the pediatrician talk with parents about infant development, highlighting new skills and acquisitions along with the potential demanding issues surrounding it.

Overall, in the light of parental needs and potential difficulties faced by parents of growth-vulnerable infants, and based on evidence of VF effectiveness in supporting parenting and child socio-emotional growth, we designed a case study to illustrate the application of a specific VF intervention designed for the pediatric care context, in order to support the parents of an SGA infant for the first 18 months of the infant’s life.

## 2. Methods

### 2.1. Case Illustration

The present case study illustrates the application of the innovative protocol of VF intervention, PC-VIT [35,36], across the first year of a child’s life with the family of an SGA infant. The case reports on a family belonging to a non-referred healthy group of primiparous parent–infant dyads attending a pediatric primary care community center, located in Pordenone, in the north of Italy. Both parents had an upper-intermediate level of education and were working full-time. Pregnancy was healthy and delivery was spontaneous at 41 + 5 gestational weeks. The baby was born weighing 3130 g (below the tenth percentile for gestational age). During the first year, growth was constant, but weight and length ranged from the third to the tenth percentile for gestational age. The parents came to the first visit and, during a clinical interview focused on newborn development, they reported high levels of anxiety and worry concerning infant physical growth and the several episodes of crying and psychomotor agitation. Based on this clinical observation, PC-VIT was applied by a pediatrician and psychotherapist in order to address parental worries concerning a child’s weight and growth. Namely, the aim of the intervention was to sustain parents in the emergence of their new parental abilities; specifically, parents were encouraged and modelled to develop mentalizing attitudes toward their child’s behaviors.

### 2.2. The Primary Care-Video Intervention Therapy

PC-VIT was proposed during the first pediatric visit, between 15 and 30 days after birth; parents were invited to receive VF consultations about physical and mental health along the typical content of well-baby visits. During subsequent health report sessions, parents and infants were recorded for about five minutes during face-to-face interaction. Shortly after registration, the pediatrician reviewed and commented on the video together with the parents. A specificity of PC-VIT is that each session is focused on a specific developmental milestone (see Table 1), which is translated into different stratagems proposed for family interaction. Indeed, VF was focused on the specific developmental challenges faced by the family at each specific time point or anticipating upcoming ones. In the first session, parents were asked to free play with their baby. In the second session, face-to-face triadic exchange was proposed, serving for joint work on affective match and mismatch episodes. In the third session, parents were asked to feed their baby; here, a relational focus on the child’s feeding is introduced. In the fourth session, parents were asked to separate from their baby for a very short time; this allows for focus to be on both parents’ and toddlers’ attitudes toward child autonomy and separation. The last two sessions were centered on reading a book together (fifth session) and on discipline (sixth session).

The general structure of each PC-VIT visit is summarized in Table 2. First, the pediatrician shows a selected part of the video (Step 1). Second, parents are encouraged to share what caught their attention (Step 2). Then, the pediatrician points out a series of positive interactive moments visible in the video and shares the reasons for regarding them as positive. In addition, the pediatrician and parents reflect together on one or more new actions that can be implemented at home to translate positive moments into routine nurturing exchanges (Step 3). Last, the pediatrician summarizes the main points elaborated in the session (Step 4).

### 2.3. Analysis of Parental Discourse

To provide an overview of potential changes in parental narrations throughout the intervention, we longitudinally analyzed the quantity of parental discourse during the PC-VIT sessions. Parental narratives constitute a powerful indicator of their thoughts, worries, and emotional states with respect to a child’ development and health. In addition, they provide the possibility to explore parental engagement in the intervention in terms of increased versus decreased verbal production. To investigate patterns of parental discourse, we first identified specific thematic areas referring to the following: (a) General themes of pediatric visits and (b) targets of PC-VIT. In particular, the first area defines the infant’s physical growth (e.g., “I used to breastfeed every three hours”; “I should remember vitamin D”). The second area identifies discourse on “mental health and developmental skills” (e.g., “He turns, we find him in all positions on the bed”). Then, we selected four areas to describe the main goals of PC-VIT. Namely, two areas fit discourse related to parenting (e.g., “We’re parents, so we try! … everything is still new for us”; “We have the task of encouraging his personality”) and parental worries related to the child’s growth (e.g., “We panicked because the banana’s slice was too big”; “Is it true that his weight at six months should be double of his birth weight?”). The last two areas focus on parental discourse identifying the child as a psychological agent. We called them child’s agency (e.g., “I see, he tries to communicate his needs”; “He is very exacting”) and child regulatory abilities (e.g., “He wants the body contact to calm down”; “For some days he has been waking up uneasily, I wonder if it might be the teeth”). Each PC-VIT session was entirely videotaped and transcribed verbatim. A fixed video camera was used placed at the wall, in front of the setting for video recording an VF, at about 1.5 m distance. From the initial transcription, text was divided into sentences, normalized removing punctuation (e.g., ellipses) and were separated for mother and father verbal productions. Then, all non-clear verbal expressions and/or sentences with no contents were removed. The remaining maternal and paternal sentences were manually gathered according to the six above-mentioned categories. We performed preliminary analyses of frequencies to quantify the amount of text (sentences) produced by each parent across each thematic area and along the six PC-VIT sessions.

## 3. Results

### 3.1. Development of Parental Discourse

**Figure 1:**
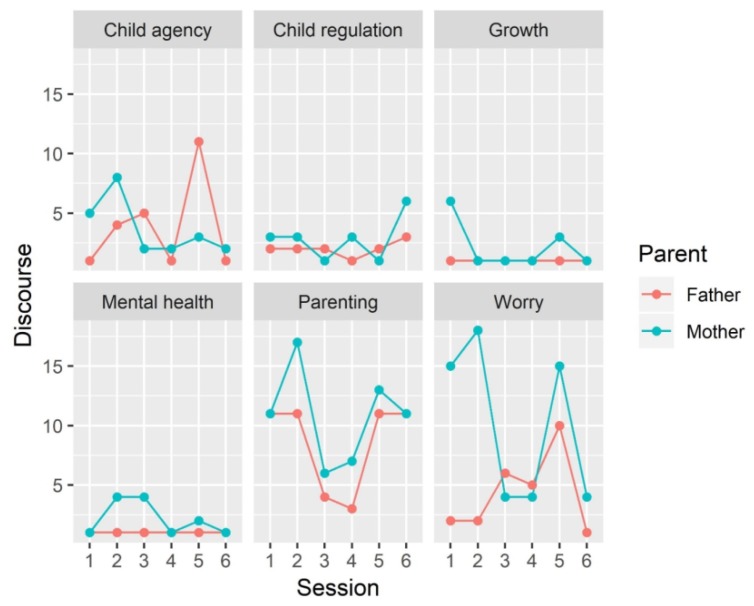
Trends in parental discourse across sessions for selected thematic areas.

Figure 1 graphically depicts the rate of change across time in the quantity of parental discourse from the first well-baby visit, in the postpartum period, to the eighteenth month of the child’s life. Through visual inspection, one can observe that areas eliciting the most maternal and paternal verbal expressions refer to parenting, parental worry, and child’s agency. In particular, “parenting” and “parental worry” texts were present from the beginning of the intervention. On the contrary, overall, less discourse was produced referring to the child’s physical growth. That might be due to the structure of a PC-VIT session, where in the first part of the visit the child’s medical examination takes place. Generally, the parents used that moment for requests and clarifications related to their son’s development.

Regarding discourse development along the intervention, maternal worry about her child’s growth was highly reported in the first three months of the child’s life; then, after a decrease in the third and fourth sessions, it increased again at 12 months.

Another interesting point is that, progressively, the parents reflected one another in the quantity of their discourse related to parental worries. Namely, across sessions, higher father involvement emerged, whereas maternal verbal production decreased, so that from the third session, the parents displayed a very similar pattern of discourse, remaining stable for the rest of the intervention. Another interesting outcome that emerged through our observation of parental narratives was the possibility for the parents to reflect about their son as an interactive agent. Notably, the parental focus on the child’s agency appeared to be present from the first session, whereas attention to the child’s emotion and state regulation appeared to emerge in parents’ discourses around the fifth session. As expected, parental mentalization of the child’s internal states required more effort than a focus on the child’s behavior. This increased attention to the child’s regulation around the sixth month of the child’s life might represent that, along the intervention, a shift of focus from behaviors to the child’s internal states took place.

### 3.2. Clinical Vignette: Eating Together (Third Session)

To give the readers a close view of PC-VIT, we present a clinical vignette to directly observe the application of PC-VIT principles within a single session. Parental worries about the growth are likely to become particularly salient along the weaning process, where parents of SGA infants might underestimate the infant’s competencies. Weaning truly matters to parents in general, representing a turning point in a child’s nutrition and autonomy. This is likely to be even truer for parents of children with growth vulnerability. We present the third pediatric session (around the sixth month of life). Generally, at this time point, parents are asked to feed their baby with a banana as the interactive stratagem that elicits discussion about feeding and weaning. As follows, we provide some extracts of the VF session to observe the interactive dynamics between the pediatrician and parents during VF.

In this first extract, the pediatrician explores parental feedback about the video (Step 2). He provides an emotionally available relational context allowing the mother to report on her worries and sustains maternal comments on the infant’s activity:

Pediatrician: *So, we saw this little part [of the video], what do you think?*

Dad: *That he is very active.*

Mom: *uhm… (punted)*

Pediatrician: *(smile at the mother) … try not to focus on the piece of banana … [the pediatrician refers to previous mother’s comments on the fear of suffocation].*

Mom: *He acts, he’s interested, he wants to experience something new, he wants to touch it.*

Pediatrician: *Yes! There you see this, how he puts his hands, he wants to try, to smell. Beyond the fear, which is a very common fear most of parents have … this fear might limit your possibility to look at how your child is growing up. As we said, you can start placing some smashed food on the table or on the plate and then you increase, you will, you will be reassured seeing that he is capable… he is programmed for this!*

Then, the pediatrician comments on the feeding interaction with parents (Step 3). He starts pointing out the infant’s interest and agency toward this new experience. By showing the child’s abilities, he aims at promoting a more positive and less worried point of observation. Additionally, he accepts and validate the parents’ worries, giving reassurance.

Pediatrician: *Here, look at how interested he is! Did you see how he hooked [the banana slice]?*

Mom: *We panicked a bit, the slice was too big!*

Pediatrician: *Okay it’s normal… Look here, is he nibbling it?*

Mom: *Yes, yes!*

Pediatrician: *Ok, here he brings the banana, can you see? Did you see that he feeds? Oh, how interested he is in this new thing! Well, what we have here? He takes [the banana], so he has his own initiative too, he puts a little of control in this thing, unlike [what he can do with] the teaspoon.*

Dad: *Yes, there he is passive*

Pediatrician: *With the teaspoon it’s just right to the mouth and stop! That’s also okay, but it might not be just that! Here (indicating the monitor with the baby holding the banana) he sees it, it has a color, a smell, he takes it, he hunts it. So, these two things can go a bit together: a little you two taking control, a little he does.*

Mom: *Maybe, to relieve this anxiety a bit… we can start with a small piece of banana! Here, I had a little bit of anxiety, but for example last night it was a smaller piece and I felt calmer, I crushed it a bit*

Dad (to the mother): *I see, this is my concern too. But if the doctor says no, I think there is no danger*

Pediatrician: *No, absolutely. Not with such soft things.*

Then, the pediatrician suggests replicating the interactive experience of feeding and eating at home (Step 3) and gives some general advice.

Pediatrician: *Also, eating together is a great idea, because he learns a lot by looking. If he is the only one who eats, and only eats with the spoon, he learns a little: just to receive the spoon.*

Dad: *So, you say, while we eat, can we also give him something?*

Pediatrician: *Give him some small pieces, some sauce…*

Dad: *Bread?*

Pediatrician: *A small piece of pasta, bread is good too, most of all I recommend variety. There are children who only eat only bread… but children need variety in food.*

Dad: *Ok.*

Last, the pediatrician goes back to parental worries that emerged in previous sessions and during the first part of this VF and links them with new emerging challenges for the parents: control and discipline (Step 4).

Pediatrician: *So, what do you think of him, how is he growing up? This moment of eating also reflects other areas of his growth. He is now with his own desires, initiatives, this new willingness to do something. How is this thing?*

Mom: *Oh, that show us he’s growing, he changed a lot just in a month, even in few weeks!*

Dad: *He shows us that he has his own intelligence, his intent, his desires and needs, and he tries to communicate them in his own way.*

Pediatrician: *Perfect!*

Mom: *He has his own personality, which is not easy!*

Pediatrician: *What do you mean?*

Mom: *That when he wants something you can see it; he makes you understand, like he cries.*

Pediatrician: *And, how do you stand with this new willingness, intentionality, which is emerging now?*

Overall, the clinical vignette highlights the positive impact that a family-oriented, mental health-focused pediatric visit can have on a vulnerable family. Observing VF during such a consultation enables to directly observe how PC-VIT works on the crucial issue of a child’s vulnerability. Indeed, several difficulties can emerge during this developmental stage, with the possibility to negatively impact the parent–child relationship. Using VF at this crucial point allows direct observation of how sustaining parents fosters their abilities in recognizing their child’s competences. This might reduce parental worries and potential negative practices deriving from such fears. In particular, the pediatrician used the video to show the parents their son’s interest toward food/world exploration. By doing that, he shifted the parental focus from their worry of suffocation to the child’s abilities. This took place in a non-judgmental and relational context, which allowed the parents to freely discuss between each other about potential alternative strategies to handle this turning point of child development.

## 4. Discussion

We presented the application of the innovative video-feedback protocol, the Primary Care-Video Intervention Therapy (PC-VIT), which combines a relational perspective and a focus on infant mental health and parenting, with the daily activity characterizing a pediatric setting. The intervention was delivered to a family (mother and father) of a Small for Gestational Age infant, a perinatal condition of growth vulnerability resulting in a birth weight below the tenth percentile for gestational age, with the weight ranging between the third to tenth percentile along the first year of life. The intervention was aimed at decreasing parental worries about the child’s growth and sustaining parental mentalizing abilities, intended as the parental ability of making sense of the child’s behaviors as expressing internal emotional and mental states [32]. Overall, the PC-VIT appeared to allow parents to progressively decrease worries and better recognize the child’s behaviors.

With this vulnerable family, the use of PC-VIT might result as particularly relevant in the developmental stage of weaning. Referring to the clinical vignette, a short interactive feeding sequence appears as being enough to activate parental worries concerning the infant’s feeding skills. Here, the relational and non-judgmental context of PC-VIT is aimed at allowing parents to share their emotional states while feeling understood, supported, and sustained in their initiatives. We observe that the pediatrician guidance might potentially sustain parents in shifting the attention from personal preoccupation towards the observation of infant competencies and initiatives. In this way, PC-VIT could modify both parents’ subjective experience of a delicate moment and their interactive behaviors, making them more aware of their reactions and giving more realistic views of their child [29]. An enhanced ability to recognize infant autonomy and competences during relevant milestones, such as the weaning process, might be transferred into more sensitive and attuned daily interactions. This is highly relevant for healthy socio-emotional development, since interpersonal regulation processes taking place during interactive exchange between mother and infant foster an infant’s daily learning of progressively more sophisticated autoregulation [37,38]. In this growth-vulnerable family, without timely and focused interventions, such worried parental representations might have progressively intensified, obscuring the possibility to recognize infant competencies and thus causing long-term damage to the quality of feeding interactions.

Overall, findings observed through clinical vignettes and the analysis of parental discourse might help to evidence that early application of PC-VIT has the potential to rapidly teach parents to recognize infant’s communicative skills and progressively identify his self—and emotion-regulatory abilities, so that parents become more able to speak about their son’s competences. These characteristics are fundamental for child development, nurture a secure parent–child attachment, and represent early markers of socio-cognitive development [39]. A specific feature of PC-VIT sustaining such parents’ learning processes is the temporal contingency of video recording and VF. Indeed, parents’ emotional involvement is still active during VF, which aids them to freely report on their emotional states. Second, the pediatrician can then intervene on potential misinterpretations of a child’s behavior driven by parental emotions, replacing parental fears and sense of inadequacy with new interpretations of a child’s actions that can modify parental inner states underlying negative thoughts and attitudes.

In regards to the application of VF in a pediatric context, PC-VIT seems to suit the well-baby visit setting, which appears to be a particularly valid context for early interventions in subclinical families. Indeed, within the routine activity, the pediatrician can prevent vulnerable infants from a clinical outcome, by accompanying and sustaining their parents throughout the foremost fragile developmental milestones. This kind of intervention can easily meet the need for early support in families with growth-vulnerable infants, who are unlikely to receive tailored follow-up care despite their known vulnerability [1,9]. Besides, the timely identification of developmental fragilities in socio-emotional development (as well as early signs of parental difficulties or fatigue), and the development of tailored strategies of intervention before clear clinical manifestations might positively impact on health costs that may occur from later interventions. Further, this timely interventions may limit the overuse of health care resources, which characterize parents perceiving their infants as vulnerable [40].

The study has some limitations. First, the lack of external measure to assess parental emotional state along the intervention and their involvement, as well as their change in child-care behaviors, strongly limit clear interpretation and the generalization of findings. In addition, with the current findings it is not possible to disentangle the positive effect of the intervention to the decrease of parental worries due to child healthy development. Future studies comparing PC-VIT intervention and typical well-baby visits are warranted to detail the investigation and assess for the potential positive effects on the socio-emotional development, or on the parental relationship as directly associated to the effectiveness of the intervention. Beyond these limitations, our preliminary findings support the encouraged shift in pediatric care towards more family-centered and mental health-focused approaches [41]. Pediatric health care providers have been called upon to develop strategies enhancing parent–child interactions [42]. Indeed, the present case study firstly shows that PC-VIT might provide an innovative answer to this point, allowing the pediatrician to address a focus on familiar and relational issues surrounding each child’s milestones and to promote an integrated approach to child development, where socio-emotional health is sustained along with physical growth.

## 5. Conclusions

Parenting is challenged by SGA vulnerability increasing worries about physical growth that might influence the healthy development of a parent–child relationship. Video-feedback shows the powerful opportunity to limit the impact of SGA growth vulnerability on parent–child and socio-emotional development by sustaining parental engagement and mentalization.

The exemplification provided by this case study serves as example to generalize PC-VIT protocols to all these contexts of growth vulnerability or perinatal fragility that do not require immediate clinical interventions, but rather would benefit from timely preventive and supporting programs sustaining socio-emotional development. Besides, our case study allows to reflect upon the importance for pediatric general health providers to promote an integrated view of child development, implementing effective ad-hoc strategies to monitor mental health, by sustaining emotional abilities and nurturing healthy parent–child relationships. Indeed, we hope this study to be meaningful for pediatricians and general health providers in order to be aware of the developmental risk of SGA children and of the challenges that parenting in these contexts undergoes. 

## Figures and Tables

**Table 1:** PC-VIT structure.

PC-VIT Session	Theme	Task
1 month	Touch and Cry	Free contact
3 months	Affective matching/Descriptive language	Face to face
6 months	Feeding	Eating together
8 months	Separation and Autonomy	Separation procedure
12 months	Reading	Reading together
18 months	Limit setting	Don’t care procedure

Note: PC-VIT = primary care-video intervention therapy.

**Table 2:** PC-VIT in the pediatric visit framework.

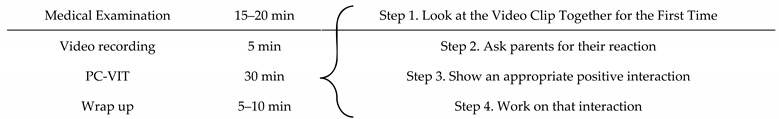

Note: PC-VIT = primary care-video intervention therapy.
